# A comprehensive review on the application of semiconducting materials in the degradation of effluents and water splitting

**DOI:** 10.1007/s11356-023-31353-3

**Published:** 2023-12-23

**Authors:** Muhammed A. Mahmoud, Bandar R. Alsehli, Mohammed T. Alotaibi, Mohamed Hosni, Ahmed Shahat

**Affiliations:** 1 Department of Physics, Faculty of Science, Suez University, Suez, 43518 Egypt; 2 Department of Chemistry, Faculty of Science, Taibah University, 30002 Al-Madinah Al-Munawarah, Saudi Arabia; 3 Department of Chemistry, Turabah University College, Taif University, P.O. Box 11099, 21944 Taif, Saudi Arabia; 4 Center for Applied Research On the Environment and Sustainability, The American University in Cairo, Cairo, 11835 Egypt; 5 Chemistry Department, Faculty of Science, Suez University, Suez, 43518 Egypt

**Keywords:** Semiconducting materials, Piezo-photocatalysis, Photocatalytic, Water pollution, Renewable energy

## Abstract

In this comprehensive review article, we delve into the critical intersection of environmental science and materials science. The introduction sets the stage by emphasizing the global water shortage crisis and the dire consequences of untreated effluents on ecosystems and human health. As we progress into the second section, we embark on an intricate exploration of piezoelectric and photocatalytic principles, illuminating their significance in wastewater treatment and sustainable energy production. The heart of our review is dedicated to a detailed analysis of the detrimental impacts of effluents on human health, underscoring the urgency of effective treatment methods. We dissected three key materials in the realm of piezo-photocatalysis: ZnO-based materials, BaTiO_3_-based materials, and bismuth-doped materials. Each material is scrutinized for its unique properties and applications in the removal of pollutants from wastewater, offering a comprehensive understanding of their potential to address this critical issue. Furthermore, our exploration extends to the realm of hydrogen production, where we discuss various types of hydrogen and the role of piezo-photocatalysis in generating clean and sustainable hydrogen. By illuminating the synergistic potential of these advanced materials and technologies, we pave the way for innovative solutions to the pressing challenges of water pollution and renewable energy production. This review article not only serves as a valuable resource for researchers and scholars in the fields of material science and environmental engineering but also underscores the pivotal role of interdisciplinary approaches in addressing complex global issues.

## Introduction

### Overview of wastewater treatment challenges

Water is considered the main resource for life for all creatures, but it is becoming scarce, and due to the development and urbanization natural sources have been degraded, and this is becoming the major challenges of the current era (Ahmadipour et al. 2021; Khan Rind et al. 2023a, b; Mishra et al. 2021; Pang et al. 2023). The acceleration in business and urbanization has undoubtedly brought improvement in many section of life; also, the production rate has been boosted to the highest levels, that also has a downside that production rate produced a tremendous amount of effluents into the atmosphere, resulting in hazardous effects in the environment such as air pollution, climate change, and depletion of the ozone layer, because of its complex composition, limited biodegradability, and high toxicity (Adane et al. 2021; Ammar et al. 2023; Liu et al. 2021a, b, c, d; Zhang et al. 2023; Zhi et al. 2021). It was estimated by UNESCO AQUASTAT that 56% of fresh water worldwide is used in the industry, domestic, and agriculture needs. Additionally, around 70% of the sewage is released into the environment without being treated first (Peramune et al. 2022). The most used common methods in the degradation of effluents may have some downsides, such as being relatively expensive and may introduce secondary pollution to the ecosystem, in addition to some processes may require a longer processing period, but not removing all the contaminants. Thus, new efficient methods shall be considered for the future (Zhang et al. 2023).

The composition of sewage is seriously complicated due to containing numerous detrimental compounds and functional groups (Ahmadipour et al. 2020a, b; Pang et al. 2022; Peramune et al. 2022). Artificial colors, antibiotics, and heavy metals are considered the most common effluents found in sewage (Adane et al. 2021; Ahmadipour et al. 2020a, b; Peramune et al. 2022). Artificial colors are used in numerous industries, such as fabric, coloring, beauty products, plastics, drug-related, and imaging (Ardani et al. 2022; Tuzen et al. 2023). These businesses consume around 100,000 distinct types of artificial dyes. There are more than 700,000 tons of dyes are produced each year (Ardani et al. 2023; Nur et al. 2022). The textile industry is primarily responsible for the extensive use and discharge of colors in the sewage; it produces around 15% (100,000 tons) of the produced dyes resulting from the dyeing processes into the fresh-water streams, contributing to the worldwide water pollution between 17 and 20% (Donkadokula et al. 2020; Nur et al. 2022). The most part of the azo group dyes (60–70%) is used in this industry, with releasing between 15 and 20% of the total dyes used into the ecosystem, causing hazardous effects (Donkadokula et al. 2020). Most of these dyes that are discharged can cause anemia, neurological disorders, toxic, and carcinogenic effects; it may also be resistant to microbial degradation, and under anaerobic degradation they may form carcinogenic compounds (Donkadokula et al. 2020; Waghchaure et al. 2022). In addition, these colors reduce the amount of sunlight that reaches the water, which has a hazardous impact on the aquatic organisms (Waghchaure et al. 2022).

Antibacterial drugs have been extensively used to enhance the growth of animals and to protect humans against microbe infections (Zhu et al. 2021). The everyday usage of antibacterial drugs has increased from about 21.1 to 34.8 billion tons, from 2000 to 2015. Also, projections suggest that the usage will rise by 67% by the year 2030 with the growth mainly be in China, the USA, India, Brazil, and Germany (Zhu et al. 2021). Furthermore, it is considered that 100,000 to 200,000 tons of antibiotics is the global consumption; these antibiotics can be detected from hospital sewage and may not be degraded in wastewater treatment plants. Some articles recorded the detection of 39 distinct antibiotics belonging to 10 different classes (Omuferen et al. 2022). The most detected antibiotic classes are sulfonamides, macrolides, trimethoprim, quinolones, and tetracyclines (Omuferen et al. 2022). A significant amount of antibacterial drugs (approximately 40–90%) is released into the waterways and the soil as unaltered drugs or primary metabolites (Cheng et al. 2018). Despite antibiotics cured humans and animals from deadly bacterial infections, their presence in the sewage may lead to the proliferation of antibiotic-resistant bacteria that poses a serious threat to the people and animals’ health and the efficiency of this medication (Wei et al. 2020). They also can cause severe health consequences, such as vomiting, nausea, acute renal failure, and diarrhea (Masekela et al. 2023a, b).

**Fig. 1:**
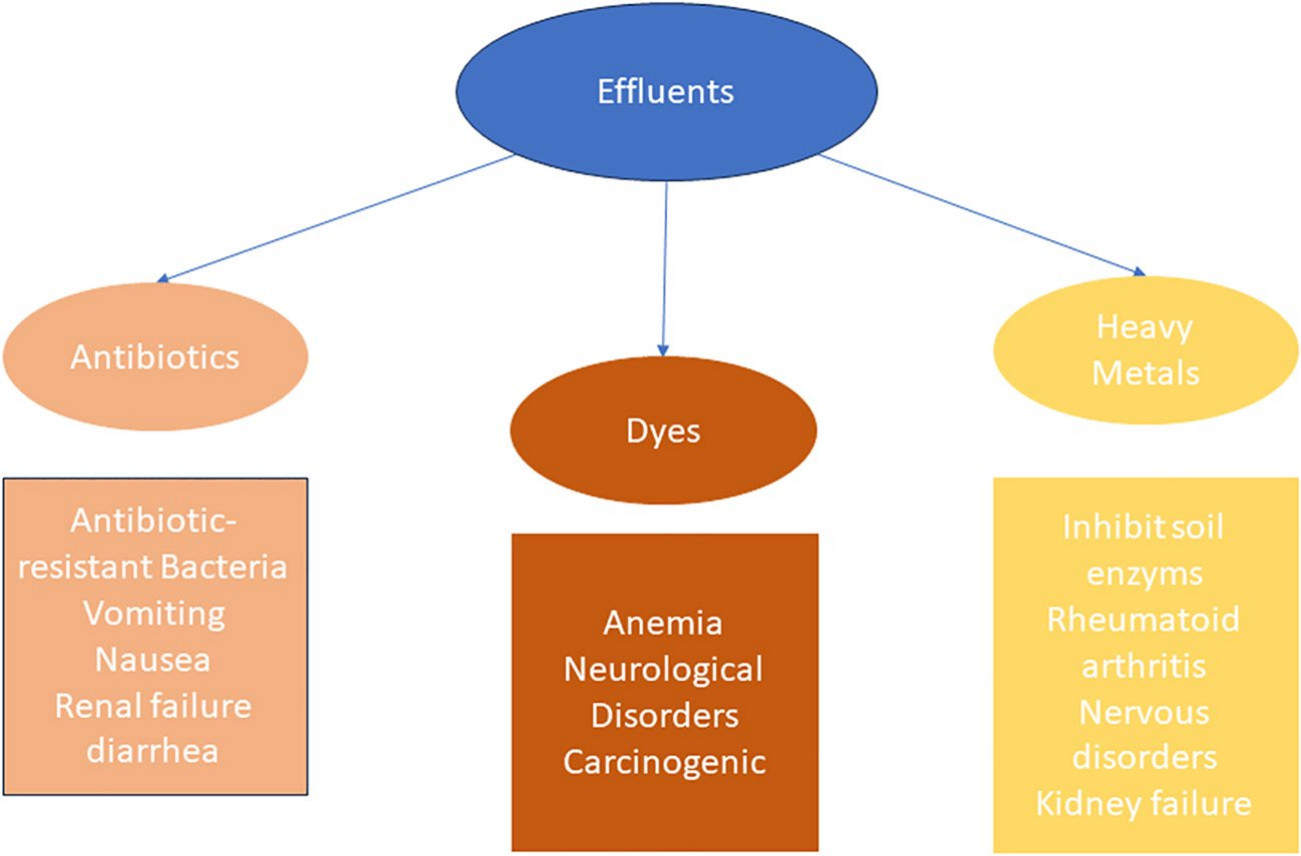
The negative effects of different effluents

Moreover, heavy metals have been detected in sewage due to the expansion of production in some areas including electrodeposition, batteries, insecticides, mining, artificial silk industry, metal cleaning, leather industry, fabric industry, petroleum-based chemicals, paper production, and electrochemical deposition (Chai et al. 2021; Iqbal et al. 2023; Qasem et al. 2021; Shrestha et al. 2021). Lead (Pb), zinc (Zn), mercury (Hg), nickel (Ni), cadmium (Cd), copper (Cu), chromium (Cr), and arsenic (As) are considered the most detected in sewage (Chai et al. 2021; Qasem et al. 2021; Shrestha et al. 2021). Humans cannot metabolize these heavy metals, which pose a risk to the public health that results from their buildup in the soft tissues (Chai et al. 2021; Iftikhar et al. 2023; Khan Rind et al. 2023a, b). Heavy metals, like copper, mercury, and chromium, have harmful impacts on both human health and the environment. Cu ions, for instance, can harm the liver, disrupt sleep, and inhibit soil enzymes. Hg can cause rheumatoid arthritis, nervous and circulatory disorders, and harm aquatic systems, while Cr exposure can Pb to symptoms like headache, diarrhea, and nausea. Cu and Pb poisoning can cause liver illness, anemia, muscle impairment, kidney failure, and damage to the infant brain (S. Y. Cheng et al. 2019) (Fig. 1).

### The role of catalytic materials in effluent degradation

Different methods were used in the degradation of effluents, such as adsorption (Chai et al. 2021), coagulation (Zhao et al. 2021a, b), sedimentation (Raeesh et al. 2023), photocatalysis (Sinar Mashuri et al. 2020), biodegradation (Wang et al. 2023a, b), and piezocatalysis (Mondal et al. 2022).

Conventional methods for treating textile wastewater, including sedimentation, chemical flocculation and coagulation, filtration, and aeration, have demonstrated some effectiveness in removing dyes from the effluent. However, these methods have several drawbacks, such as the generation of toxic by-products, high energy consumption, unpleasant odors, and the need for a large treatment area. These limitations have prompted researchers to explore more efficient technologies that can enhance the quality of textile wastewater treatment and reduce the environmental impact of the final discharge. As a result, there has been a growing interest in developing advanced industrial processes for treating textile wastewater using more effective techniques over the past few years (Al-Gheethi et al. 2022).

Nanomaterials, including adsorbents and photocatalysts, are extensively used to remove complex wastewater contaminants, such as heavy metals, organic pollution, and radionuclides. This is due to their chemically stable, small size, rapid electron transport, large surface area, and multiple active sites (Chen et al. 2022a, b; Lanjwani et al. 2023; Zhao et al. 2018). The primary nanomaterials utilized for environmental pollutant remediation include carbon-based materials like carbon nanotubes and graphene oxides; nano-zerovalent iron (nZVI); metals and metal oxides such as silver (Ag), zinc oxide (ZnO), titanium dioxide (TiO_2_), and copper(II) oxide (CuO); organic frameworks (COFs and MOFs); and transition metal nitrides, carbides, or carbonitrides (MXenes). Carbon-based materials are frequently utilized as electrodes, adsorbents, and photocatalysts for efficient contaminant removal due to their large specific surface area, optical transmittance, and high current density. Additionally, nZVI has been found to be an effective option for the degradation of organic pollutants owing to its large surface area and reactivity (Li et al. 2023a, b, c). Of the numerous nanomaterials available, certain types possess piezoelectric characteristics that enable the conversion of mechanical energy into chemical energy (Li et al. 2023a, b, c). Piezoelectric materials have emerged as a desirable alternative to achieve enhanced catalytic activity. Typically, these materials find widespread use in a range of applications, including energy generation, sensor devices, and charge storage. By utilizing these functional materials, it is now possible to make headway in catalytic processes using clean energy sources such as ultrasonic waves, mechanical vibration, and pressure (Mondal et al. 2022).

### Introduction to piezocatalytic materials

In 1880, the Curie brothers were the first to observe the occurrence of piezoelectricity, which involves generating electricity through the application of mechanical pressure on various materials (Qian et al. 2020). The origin of the term “piezoelectricity” can be traced back to two Greek words: “piezo,” which means to apply pressure or compress, and “electron,” which refers to the electrostatic charge of amber. Piezoelectricity is the electrical energy produced when a piezoelectric material is subjected to deformation. This effect is a molecular phenomenon that can be observed at the macroscopic level (Dahiya and Valle 2013). Substances that demonstrate piezoelectricity are known as piezoelectric materials. These materials produce an electrical charge in reaction to an applied force, which is called the direct piezoelectric effect, and undergo mechanical deformation when exposed to an applied electric field, which is called the converse piezoelectric effect (Bowen et al. 2014; Dahiya et al. 2013; Starr and Wang 2015). Piezoelectricity arises from the asymmetrical arrangement of positive and negative electric charges within a material’s unit cell, which does not possess a center of symmetry (Liang et al. 2019; Shi et al. 2016). When a piezoelectric substance is subjected to external pressure or mechanical oscillations, the movement of ions within the material leads to a change in the dipole moment of the unit cell, resulting in a net electrical charge. This phenomenon creates a piezoelectric potential across the material (Katsouras et al. 2016; Liang et al. 2019; Marino and Becker 1970).

Quartz and tourmaline were considered being natural piezomaterial (Liang et al. 2019). On the other hand, BaTiO_3_ (Khalal et al. 1999), PbTiO_3_ (Yourdkhani and Caruntu 2011), Pb(Zr,Ti)O_3_ (Sawaguchi 1953), KNbO_3_ (Wan et al. 2012), LiNbO_3_ (Edon et al. 2009), and LiTaO_3_ (Smith and Welsh 2003) are considered synthetic piezoelectric ceramics. While polyvinylidene fluoride (PVDF), polyparaxylene, poly-bis(chloromethyl)oxetane (BCMO), aromatic polyamides, polysulfone, and polyvinyl fluoride (PVF) are synthetic piezoelectric polymers (Kawai 1969; Villar et al. 2012). MoS_2_, BaTiO_3_, and BiFeO_3_ are considered the most efficient piezocatalytic material used in wastewater treatment (Mondal et al. 2022). Meanwhile, the development of hybrid and adaptable polymer-nanocomposites is expected to play a crucial role in regulating the excessive use of dangerous pollutants, thus reducing their negative impact on the environment. Furthermore, it will underscore the significance of polymeric encapsulation of piezocatalysts (Mohammadpourfazeli et al. 2023; Mondal et al. 2022).

## Piezocatalytic mechanisms

### Understanding piezoeffect and its application in catalysis

Piezoelectric materials are classified as smart materials that generate electrical charges when subjected to mechanical vibrations. These smart materials also display an inverse piezoelectric effect, meaning that they can produce mechanical vibrations when exposed to an electric field (Masekela et al. 2023a, b). The intrinsic electric field present in materials can influence the chemical reactions occurring on their surfaces (Liang et al. 2019). Piezocatalysis is an efficient method for treating sewage that converts the ambient mechanical vibration energy to electrochemical energy in order to start chemical reactions. During the piezocatalysis process, the mechanical vibration energy induces a polarization in the piezoelectric material, which generates a positive and negative charge output (Mondal et al. 2022; Zhang et al. 2023). Subsequently, these piezoelectric-induced charges can react with dissolved oxygen and hydroxyl groups in the solution, leading to the production of several active species (hydroxyl and superoxide radicals) that possess potent redox abilities, which can effectively treat wastewater by breaking it down to simpler and less toxic byproducts (Mondal et al. 2022; Zhang et al. 2023).

### Mechanisms of piezocatalytic materials in effluent degradation

The study conducted by Hong et al. was significant as it marked the first instance of the publication of research findings related to the generation of vibration-induced charges from ZnO microfibers and BaTiO_3_ microdendrites, which led to the direct splitting of water molecules (Hong et al. 2010).

Previously published reports have presented reactions from (1) to (12) demonstrating the production of free charges and their involvement in redox reactions during the piezocatalytic process (Hong et al. 2012).

1 $${BaTiO}_{3}+Vibration \to {BaTiO}_{3}\left({e}^{-}+{h}^{+}\right)$$

Anode (negatively charged sides of the BaTiO3):

2 $$4{e}^{-}+4{H}_{2}O \to 4{OH}^{-}+4{H}^{.}$$

3 $$4{H}^{.} \to 2{H}_{2}$$

Overall:

4 $$4{e}^{-}+4{H}_{2}O \to 4O{H}^{-}+2{H}_{2}$$

Cathode (positively charged sides of the BaTiO_3_):

5 $$4{OH}^{-}\to 4{e}^{-}+{4}^{.}OH$$

6 $$2{(}^{.}OH+{ }^{.}OH)\to 2{H}_{2}O+2{O}^{.}$$

7 $$2{O}^{.}\to {O}_{2}$$

Overall:

8 $$4{OH}^{-}\to 4{e}^{-}+2{H}_{2}O+{O}_{2}$$

Net reaction to water decomposition:

9 $$2{H}_{2}O\to 2{H}_{2}+{O}_{2}$$

Dye decomposition:

10 $${}^.OH+dye\rightarrow degradation\;products\;of\;dye$$

11 $$e^-+dye\rightarrow degradation\;products\;of\;dye$$

12 $$h^++dye\rightarrow degradation\;products\;of\;dye$$

### Photocatalysis

Photocatalysis typically pertains to semiconductor photocatalysis, primarily due to the inherent characteristics of semiconductors that make them the preferred choice for most photocatalytic processes. Semiconductors possess unique energy band structures, marked by discrete energy levels, a feature that distinguishes them from other materials. Within the semiconductor framework, the highest energy band is known as the conduction band (CB), while the lowest energy band is referred to as the valence band (VB). When provided with sufficient energy, electrons within the VB can make leaps to the CB, leaving behind holes in the VB. The minimum energy required for this electron transition is defined as the band gap (*E*_g_), representing the energy difference between the lowest CB and the highest VB (Raza et al. 2021; Tasleem and Tahir 2020; Wang et al. 2022a, b, c, d, e; Zhang et al. 2022a, b).

In the context of numerous photocatalytic applications, such as hydrogen (H_2_) production, carbon dioxide (CO_2_) reduction, degradation of organic pollutants, and nitric oxide (NO) removal, the photocatalytic reaction primarily comprises three fundamental stages (Raza et al. 2021; Tasleem and Tahir 2020; Wang et al. 2022a, b, c, d, e; Zhang et al. 2022a, b).

This fundamental understanding of semiconductor band structures and their role in photocatalysis forms the basis for various environmentally significant processes (Raza et al. 2021; Tasleem and Tahir 2020; Wang et al. 2022a, b, c, d, e; Zhang et al. 2022a, b).

#### Photocatalysis mechanism

**Fig. 2:**
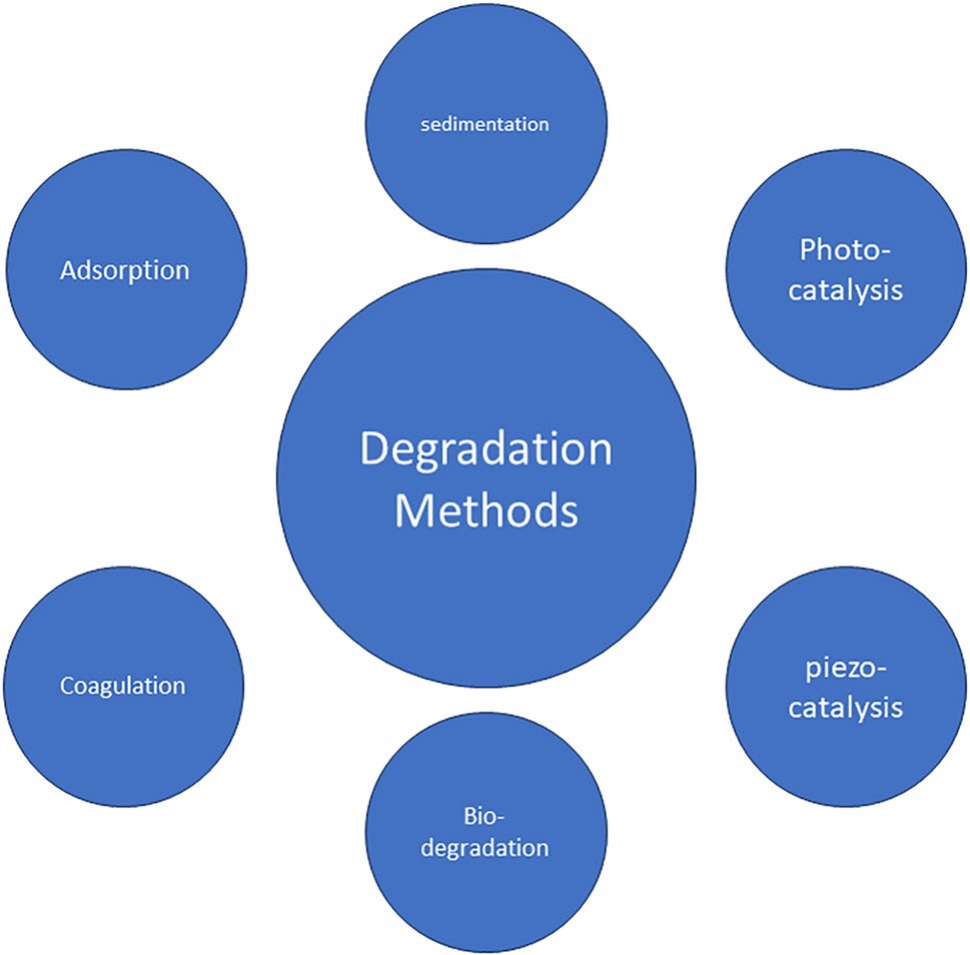
The different methods for the degradation of effluents

As shown in Fig. 2, the mechanism is demonstrated below:

- Step 1: The process begins with the absorption of light and the subsequent generation of charge carriers. When the surface of the photocatalyst is illuminated by light with energy equal to or exceeding the band gap energy of the semiconductor material, an immediate electron transition occurs, giving rise to the creation of electron–hole (*e*^−^  − *h*^+^) pairs. It is worth noting that light is typically categorized into two wavelength ranges: ultraviolet (UV) light, spanning 200–400 nm, and visible light, covering the 400–800-nm range. Notably, when the band gap energy (*E*_g_) of a semiconductor is lower than approximately 3.1 electronvolts (eV), the material can effectively absorb visible light. This ability is of great significance because visible photons constitute a major portion of sunlight, contributing to about 50% of its composition. Consequently, an ideal photocatalyst should possess the capability to efficiently absorb light—a prerequisite for initiating and sustaining photocatalytic reactions. This absorption of light serves as the initial step in the intricate process of harnessing solar energy for various environmentally significant applications (Mai et al. 2021; Raza et al. 2021).
- Step 2: The next crucial phase involves the separation and movement of these charge carriers. As light triggers the transition of electrons from the VB to the CB, it leaves behind holes in the VB. This electron–hole (*e*^−^  − h^+^) separation is a pivotal step in photocatalysis. However, it is essential to acknowledge that the recombination of these photogenerated electrons and holes is an inherent and unavoidable process. Unfortunately, this recombination can hinder the efficient utilization of charge carriers, ultimately diminishing the catalytic activity of photocatalysts (Liang et al. 2020).

To combat this challenge and maximize the efficiency of charge carrier separation and transfer within photocatalysts, numerous strategies have been devised. These methods encompass reducing particle sizes, carefully managing surface defects, exposing active sites, and increasing specific surface areas. Each of these approaches contributes to enhancing the overall effectiveness of photocatalysts by mitigating the impact of recombination and optimizing the utilization of charge carriers during the photocatalytic reaction (Liang et al. 2020).

- Step 3: The subsequent step involves surface redox reactions of the corresponding reactants. This entails the rapid transfer of electrons, capable of reduction, and holes, possessing oxidation potential, to designated reaction sites on the surface of the photocatalysts. Thermodynamically speaking, achieving successful redox reactions necessitates a precise alignment between the energy band structure of the semiconductor and the redox reaction potentials. This alignment mandates that the energy level of the CB be more negative than the reduction potential, while the energy level of the VB must be more positive than the oxidation potential. Therefore, the presence of a suitable energy band structure stands as a fundamental thermodynamic prerequisite for the initiation of photocatalytic reactions (Raza et al. 2021; Wang et al. 2022a, b, c, d, e).

Considering these critical factors, semiconductors characterized by appropriate band gap energies become indispensable for effective light absorption and robust redox capabilities. Remarkably, metal halide perovskite materials exhibit the ability to fine-tune their band gap energies, making them exceptionally well-suited for fulfilling this essential requirement in the realm of photocatalysis (Raza et al. 2021; Wang et al. 2022a, b, c, d, e).

### Piezophoto

**Fig. 3:**
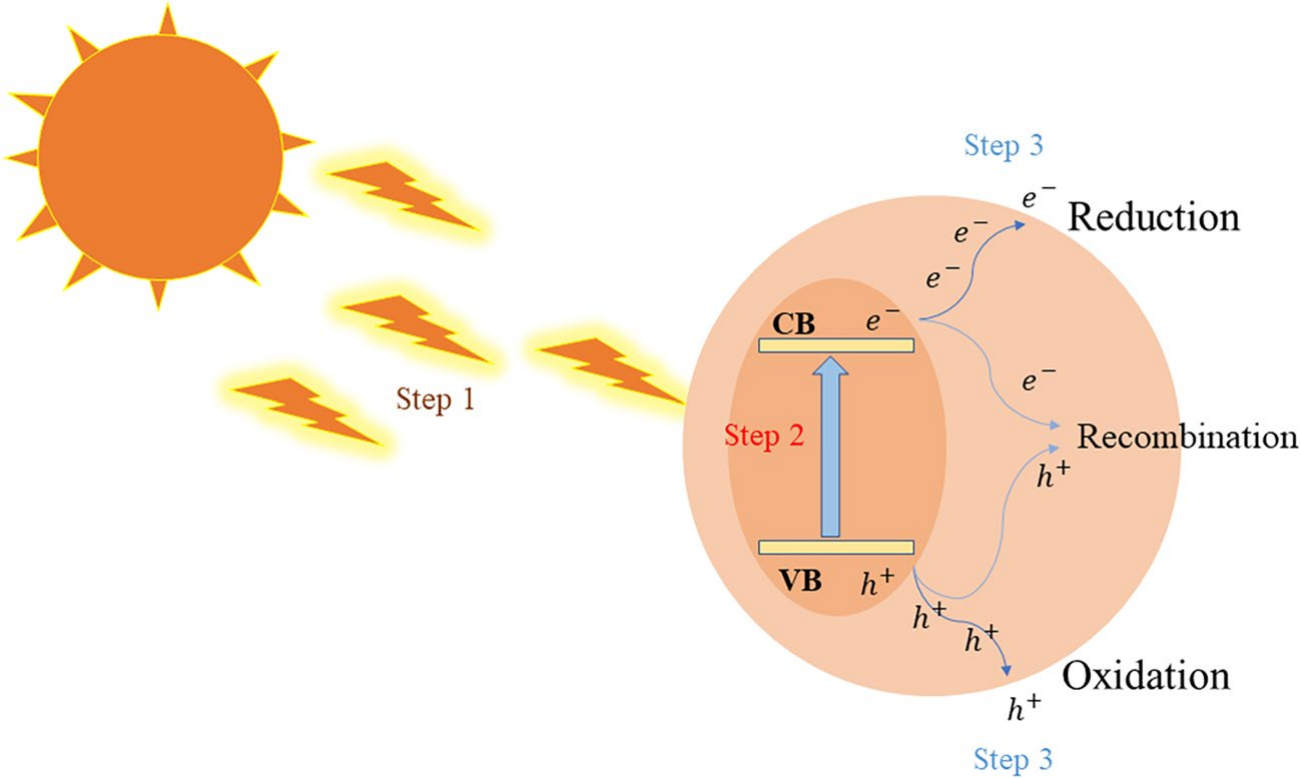
Representation of photocatalytic process, hole generation from valence energy band (VB), and electron generation from conduction energy band (CB)

Recently, there have been efforts to enhance the efficiency of photocatalytic processes by integrating the piezoelectric field of piezoelectric materials. By combining the properties of semiconducting piezoelectric materials with photon excitation, a unique effect known as piezo-photoronics arises in non-centrosymmetric semiconductors. This effect involves the coupling of piezoelectric, semiconductor, and photonic properties, and can be utilized to control the separation, transport, and recombination of charges at the interface between the semiconductor and piezoelectric materials. Consequently, piezo-photoronics can be used as a means of improving the performance of piezo-photocatalysis (Nie et al. 2021; Wang 2012) (Fig. 3).

## Effluents

### Dyes

**Fig. 4:**
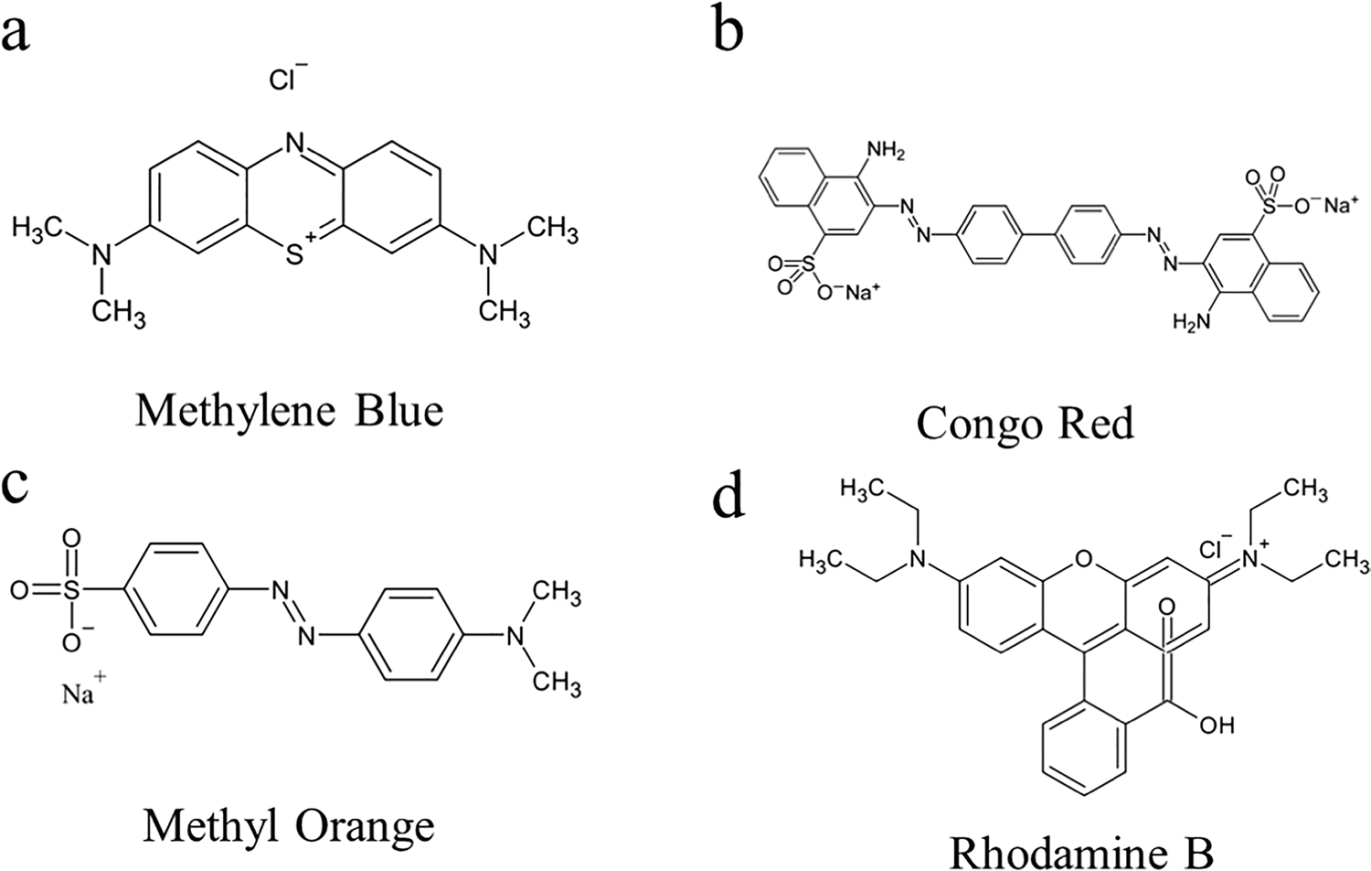
The chemical structure of several dyes

The utilization of dyestuffs in textile and other chemical engineering sectors results in the discharge of substantial quantities of highly soluble dyestuffs, thereby causing significant water pollution (Ghaemi and Safari 2018; Liu et al. 2018a, b). Some companies that use colors create effluent that is detrimental to both humans and the ecosystem, both living and non-living. As a result, residents and environmentalists are growing increasingly concerned about the presence of pigments in waterways. To reduce its detrimental effects, dye sewage should be treated before being released in an open waterway (Solayman et al. 2023). Because of their coloring, toxicity, and non-biodegradability, organic pollutants found in the fabric and coloring industry wastewaters have been one of the greatest ecological issues in the globe (Nguyen et al. 2021). In recent years, toxic, carcinogenic, and mostly non-biodegradable organic pollutants such as dyes have seriously harmed both the environment and the public well-being. Considering the expansion in fabric and coloring businesses (Sarkodie et al. 2023), rhodamine B (RhB) (Fig. 4d), known for its high toxicity, has been widely used in the textile industry, despite its reputation as a dye that cannot be easily broken down by natural processes, and has a strong color intensity (Al-Gheethi et al. 2022; Xu and Ma 2021). RhB finds its application in various industries such as the manufacturing of ballpoint pens, paints, leather goods, dye lasers, carbon sheets, stamp pad inks, crackers, and fireworks (Imam and Babamale 2020). RhB dye is categorized as a cancer-causing and nerve-damaging substance that can lead to respiratory tract infections, skin irritation, gastrointestinal discomfort, and eye infections. It has also been linked to developmental and reproductive toxicity in both animals and humans (Hamdaoui 2011). Prolonged exposure to RhB can be hazardous if inhaled or ingested, leading to liver and thyroid impairment as well as skin and eye irritation (Bhat et al. 2020). Despite being in low concentrations, RhB dye can exhibit a deadly effect on all marine life (Nguyen et al. 2021; Rafique et al. 2020; Rati et al. 2023). Similarly, Methylene Blue (MB) (Fig. 4a) is immensely used in the many industries such as cardboard and fabrics. Also, nutrition, beauty, and medical industries employ MB in a huge portion in their products (Oladoye et al. 2022), despite having some beneficial effects if taking with a medical/clinical observation in treating malaria, in the treatment of vasoplegia after transplant operation, in addition to heparin neutralization. But it can cause deadly symptoms if consumed from contaminated water (Oladoye et al. 2022). MB dye could cause a number of illnesses, including cyanosis, tissue necrosis, the development of Heinz bodies, vomiting, jaundice, shock, and an accelerated pulse rate (Oladoye et al. 2022). Also, it can block the growth in plants in addition to reducing the pigments (Oladoye et al. 2022). Another prominent anionic/acidic pigment that poses concerns to the ecosystem is Methyl Orange (MO) (Fig. 4c). Many sectors, such as the paper, foodstuff, fabric, pharmaceutical, and other research-based labs, have made substantial use of MO dye (Subbaiah Munagapati et al. 2023). If by accident, if the MO pigment gets into a person’s bloodstream, the gastrointestinal bacteria turn the pigment into an aromatic amine, which could also make people cyanosis, quadriplegic, jaundiced, throw up, and have a faster heartbeat (Subbaiah Munagapati et al. 2023). Despite the widespread use of Congo Red (CR) (Fig. 4b), this dye is inherently toxic to living organisms (Munagapati and Kim 2016; Waheed et al. 2019). Due to its intricate aromatic structure and robust chemistry and thermal stability, wastewater containing CR is a hazardous form of organic wastewater that poses a challenge for degradation and requires significant chemical oxygen (Borthakur et al. 2017; Wang et al. 2018a, b). Furthermore, when subjected to anaerobic conditions, it breaks down into benzidines, a known carcinogenic compound (Y.-Y. Chen et al. 2018; Miandad et al. 2018; Song et al. 2016).

### Antibiotics

Antibacterial agents are widely employed as effective medications and enhancers of animal growth in the domains of human healthcare, animal husbandry, and aquatic farming (Kümmerer 2009; Liu et al. 2018a, b). Antimicrobial agents are additionally discharged into aquatic ecosystems through sewage, hospital effluent, and animal excrement. The inadequate purification of wastewater containing antibiotics in sewage treatment facilities amplifies the pollution of surface water reservoirs, subsurface aquifers, and possibly potable water sources (B. M. Sharma et al. 2019). The extended existence of antimicrobial agents in the surroundings applies selective pressure on microbial populations, prompting them to generate genes that confer resistance to antibiotics, that are antibiotic resistance genes (ARGs) (Shao et al. 2018). ARGs harbored by antibiotic-resistant bacteria (ARB) may be transferred and propagated to human pathogenic microorganisms, which can disseminate from the natural milieu and pose a significant peril (Hawkey and Jones 2009). Due to its fluidity and rich microbial diversity, the aquatic ecosystem serves as the primary conduit for the dissemination of antimicrobial agents and ARGs (Yang et al. 2018; Zainab et al. 2020). The quantity of wastewater produced per hospital bed on a daily basis ranges from 40 to 120 L in developed nations, while in developing countries like Nigeria, it ranges from 2 to 50 L (Kumari et al. 2020). Antimicrobial agents are introduced into aquatic ecosystems via the release of treated wastewater and surface runoff, which have been extensively identified in lakes (Zhou et al. 2022a, b), rivers (Kong et al. 2022), and other ecosystems (Wang et al. 2021a). ARGs carried by ARB could propagate through hydraulic exchange, microbiomes, and food chains, exacerbating the threat to human health and ecological equilibrium (Adachi et al. 2013; Cabello et al. 2013). The analysis of metagenomics unveiled the existence of 383 distinct subtypes of ARGs in aquaculture ponds (Ning et al. 2022). Ciprofloxacin (CIP) is classified as a quinolone antibiotic (McShane et al. 2018), and is primarily utilized in the management of bacterial infections, particularly those affecting the urinary tract. Urinary tract infections (UTIs) are a highly prevalent global health issue, particularly among women. Of the 8 million cases reported annually, approximately 10 out of 25 women and 3 out of 25 men typically experience symptoms. In addition, CIP is recognized for its efficacy in treating spontaneous bacterial peritonitis in patients (Ajala et al. 2022). CIP is incompletely or non-metabolized in animals upon administration, resulting in the discharge of both the original compound and its metabolites into other environmental compartments, such as water bodies, during excretion (Al-Haideri et al. 2021). In addition, traditional methods of treating wastewater, such as activated sludge or up-flow anaerobic sludge blanket reactors, are constrained in their capacity to eliminate CIP (Kim et al. 2020). It has been reported that hospital wastewater may contain as much as 34 g/L of CIP, implying that CIP is a persistent compound with high environmental resilience (Diniz et al. 2021). Exposure to CIP has been associated with alterations in the antioxidant enzymes of affected organisms (Trombini et al. 2021).

Carbamazepine (CBZ) has garnered widespread recognition in the medical field for its efficacy in treating numerous disorders, including depression, epilepsy, and arrhythmia. Nevertheless, due to its extensive utilization, both the compound and its metabolites have been discovered in water bodies at levels ranging from ng/L to µg/L, resulting in various deleterious impacts on aquatic organisms (Adeyanju et al. 2022). It is projected that approximately 5 million individuals worldwide are diagnosed with epilepsy annually, with an estimated incidence rate of 49 cases per 100,000 individuals in high-income countries. In middle- and low-income nations, 139 people are diagnosed with epilepsy each year (Ajala et al. 2022). Numerous scholars have investigated the ecotoxicology of CBZ. For instance, in a Shanghai sewage treatment facility, concentrations of CBZ ranged from 230 to 1110 ng/L, while in the Yangtze River, the concentration was measured at 1090 ng/L (Chen et al. 2019). It has been reported that 100% of CBZ was detected in the rivers of Nanjing, with concentrations ranging from 0.05 to 1.6 ng/L in fish tissue and 0.2 to 6.9 ng/L in the water system (Liu et al. 2015).

Tetracyclines (TCs) are a class of broad-spectrum antibiotics that contain a phenanthrene parent nucleus, including tetracycline (TC), oxytetracycline (OTC), and doxycycline (DC). They are extensively employed in the treatment and prevention of diseases in humans, animals, and plants (Xu et al. 2021a, b). In Japan, the employment of TC antibiotics in veterinary medicine constitutes 43% of the overall usage of antibiotics (Xu et al. 2021a, b). Meanwhile, in the UK, TCs accounted for 22.1% (Cheng et al. 2020). According to information released by the U.S. Food and Drug Administration (FDA) in 2015, the yearly sales of TCs antibiotics in the USA totaled 6.514 × 106 kg, representing 44% of all veterinary pharmaceuticals (Aidara-Kane et al. 2018). Nonetheless, TC, the most widely utilized member of the TCs, cannot be entirely assimilated by humans and animals, resulting in the excretion of approximately 50–80% of the residues into the environment via fecal matter (Mahamallik et al. 2015). In a study by Samaraweera et al. (2019), it was discovered that concentrations of TCs in influent water at wastewater treatment plants could reach up to 435 ng/L. TC is also accumulated in sludge. Maruzani et al. (Wang et al. 2018a, b) investigated the sludge from a wastewater treatment plant in the United Kingdom and detected TC levels as high as 160 ng/g in activated sludge. Additionally, the concentrations of TCs, including TC, OTC, and DC, in medical wastewater were considerably elevated, with levels reaching 2596.5 ng/L, 345.6 ng/L, and 670 ng/L, respectively. Compounding the issue is the fact that, owing to its polarity and polyionic groups, TC remnants can readily disperse into other environmental media, such as water bodies and soil (Huang and Liu 2023).

The existence of antimicrobial agents in aquatic ecosystems has contributed to the development of bacterial resistance genes (Zainab et al. 2020). The presence of antimicrobial remnants in aquatic ecosystems also has repercussions on non-bacterial aquatic biota, such as fish, copepods, microalgae, and macrophytes (Zainab et al. 2020).

## The piezo-photocatalytic ZnO-based materials

In the seventeenth century, Zn was rediscovered as a metal and the term “zinc” gained widespread recognition. Scientists found a way to condense the vapors and use them for smelting in an environment free from oxygen exposure (Pande 1996). Eventually, zinc was included in the periodic table (Partington 1989). Zinc is typically not found in its pure form in nature, but rather in combination with other elements, such as oxygen or sulfur. One of the most notable forms of Zn is ZnO, which possesses exceptional physical and chemical properties that make it an extremely functional material. Its excellent chemical stability, superior electrochemical coupling coefficients, outstanding radiation absorption, and remarkable photostability all contribute to its significance as a functional material (Ali et al. 2023). ZnO was first utilized for its semiconducting properties in the 1920s as radio signal rectifiers for do-it-yourself radio sets. Since then, research on ZnO has expanded significantly over the last two decades, making it one of the most extensively studied materials. The first ZnO electron diffraction pattern was reported in 1935 (Yearian 1935). In 1954, the *n*-type properties of ZnO were initially confirmed through Hall measurements that were dependent on temperature (Harrison 1954).

ZnO is a white powder that has low solubility in water but can attach itself to acids and alkalis upon contact. It is a naturally occurring substance that can be found in zincite, which has three distinctive crystal structures: Zn blende, rock salt, and wurtzite. At room temperature and atmospheric pressure, ZnO takes on the form of hexagonal wurtzite, featuring tetrahedrally coordinated zinc cations and oxygen atoms. The crystal lattice parameters of zinc oxide are 3.25 Å and 5.20 Å. A more detailed explanation of the structure of ZnO can be found elsewhere (Lam et al. 2012; Niskanen et al. 2013; Özgür et al. 2005).

H. Lv et al. conducted an experimental study in which they mixed BaTiO_3_ with ZnO to form a composite material (Lv et al. 2023). Subsequently, they employed the electrospinning technique to fabricate Janus nanofibrous using PVDF as a substrate. The resulting Janus nanofibrous exhibited remarkable catalytic activity, as evidenced by its ability to degrade various organic pollutants, including bisphenol A (BPA), CR, MB, and tetracycline hydrochloride (TCH), with an impressive efficiency of 91.05%, 90.12%, 96.33%, and 93.65%, respectively, within a span of 1 h (Lv et al. 2023). In a study conducted by C. Zhang and colleagues (Zhang et al. 2021a, b), the highly effective combination of ZnO/CdS was utilized to successfully and completely eliminate bisphenol A in just 30 min (Zhang et al. 2021a, b). This achievement is significant because BPA is a harmful chemical that can have adverse effects on human health and the environment (Li et al. 2023; Zhang et al. 2021a, b). The use of ZnO/CdS as a method of removing BPA represents a promising advancement in the field of water purification and environmental remediation. This study highlights the potential of ZnO/CdS as a practical and efficient solution for eliminating BPA from water sources. The successful removal of BPA using ZnO/CdS could have important implications for improving water quality and protecting human health and the environment (Zhang et al. 2021a, b). Bootchanont et al. (Bootchanont et al. 2022) conducted experiments on the nanofibers of PVDF by electrospinning as matrix and ZnO/Cu as nanocomposites and studied the degradation RhB dye. In their study, A. Bootchanont and colleagues investigated the potential of using the piezoelectric polymer PVDF as a matrix for the generation of a bi-piezoelectric integrated effect, achieved through the combination of a piezoelectric semiconductor photocatalyst, ZnO/Cu (Bootchanont et al. 2022). The electrospinning technique was employed to create the PVDF nanofiber matrix, which was then combined with the ZnO/Cu nanocomposites (Bootchanont et al. 2022). The study focused on the degradation of RhB dye, a model organic pollutant that poses a significant threat to the environment and human health (Rati et al. 2023; Wang et al. 2022d). The results of the experiment showed that the combination of the PVDF matrix and ZnO/Cu nanocomposites generated a bi-piezoelectric integrated effect that was highly effective in degrading RhB dye. Remarkably, the experiment achieved complete degradation of RhB dye within 90 min (Bootchanont et al. 2022). The use of piezoelectric polymers like PVDF, in combination with piezoelectric semiconductor photocatalysts such as ZnO/Cu, represents a promising strategy for addressing environmental pollution (Bootchanont et al. 2022). The findings of this study could lead to the development of practical and effective solutions for the degradation of organic pollutants in various environmental settings, with the added benefit of harnessing piezoelectric energy. Overall, this study highlights the potential of bi-piezoelectric integrated effect generation for environmental remediation, with the impressive result of achieving complete degradation of RhB dye within 90 min (Bootchanont et al. 2022). In their research, X. Zhao and colleagues (X. Zhao et al. 2022) achieved impressive results in the removal of methyl orange dye from water using a novel ternary heterostructure PVDF/ZnO/Au (PZA) nanobrush. The study focused on the removal of 20 mg/L of MO dye, a common and harmful organic pollutant found in industrial wastewater (Zhao et al. 2022). The PZA nanobrush was tested under both solar and mechanical energy, and remarkably, the researchers were able to remove 100% of the MO dye within just 60 min. This result demonstrates the high effectiveness of the PZA nanobrush for the removal of organic pollutants from water (Zhao et al. 2022). The use of a ternary heterostructure like PVDF/ZnO/Au in the PZA nanobrush represents an innovative approach to addressing water pollution. The combination of these materials creates a highly efficient and versatile nanobrush that is effective under both solar and mechanical energy (Zhao et al. 2022). The findings of this study could have significant implications for the development of practical and effective solutions for the removal of organic pollutants from water sources (Zhao et al. 2022). Overall, the success of the PZA nanobrush in removing 100% of the methyl orange dye within 60 min under both solar and mechanical energy highlights its potential as a promising tool for environmental remediation (Zhao et al. 2022). Gao and collaborators (Gao et al. 2023a, b) succeeded in developing a nanocomposite material comprising ZnO and CuS, which effectively degrades organic pollutants using piezo-photocatalysis. In fact, the ZnO/CuS composites exhibited an impressive degradation efficiency of 85.28% for TCH within a timeframe of 60 min via piezo-photocatalysis (Gao et al. 2023a, b). Bettini et al. (Bettini et al. 2023) conducted research on the piezo-photocatalytic degradation of steroid hormones, utilizing ZnO nanostructures as the catalyst. Through their experimentation, they were able to achieve a noteworthy degradation of 50% for testosterone in a duration of approximately 45 min (Bettini et al. 2023). Ren and colleagues’ (Ren et al. 2023) innovative approach in fabricating a carbon nitride-decorated ZnO nanoarray on a three-dimensional Ni foam substrate has yielded a promising material for the degradation of MB. The material exhibited a remarkable degradation efficiency of 93.7% within 120 min, starting from an initial concentration of 10 mg/L (Ren et al. 2023). Their results suggest that this novel material has potential in various environmental applications, such as wastewater treatment and remediation of polluted water bodies. Further investigations could reveal the full potential of this material in addressing the persistent challenge of water pollution (Ren et al. 2023) (Table 1).

**Table 1:** Performance of ZnO-based composites with different effluents

Catalyst	Catalyst dosage	Condition	Performance	Effluent	Effluent concentration (mg L^−1^)	Reference
CuS/ZnO	100 mg	200-W US, 500-W Xe lamp	100% 20 min	MB	5	Hong et al. (2016)
Zn_1−*x*_SnO_3_	2.4 cm × 2.4 cm	0.2 W 40 kHz, 300-W UV	75% 120 min	MB	4	Wang and Chang, (2016)
ZnO/TiO_2_	100 mg, 30 mg	US, 500-W mercury lamp	99% 90 min, 100% 600 min	MO, MB	20, 5	Wang et al. (2016)
Ag_2_O/tetrapod-ZnO	200 mg	200-W US, 50-W UV lamp	99% 2 min	MB	5	Sun et al. (2016)
Ag_2_S/ZnO	–	US, simulated solar light	100% 120 min	MB	1	Zhang et al. (2017)
ZnO nanorod	0.5550 g	160 W 40 kHz, 300-W Xe	45% 120 min	MO	10	Bai et al. (2019)
ZnO nanowire	–	160 W 40 kHz, UV lamp	74.9% 180 min	MB	–	Zhang et al. (2019)
ZnO nanoparticles	100 mg	120 W 40 kHz, UV lamp	90% 120 min	RhB	5	Chimupala et al. (2020)
ZnO nanosheet microspheres	–	40 kHz, 60-W UV lamp	73.5% 120 min, 86.6 120 min, 40.1% 120 min	MO, RhB, acid orange 7 (AO7)	10	Bai et al. (2020)
ZnO-Ag_8_S	–	50-W US, 250-W tungsten lamp	93.11% 120 min	RhB	479.02	Venugopal et al. (2020)
Au–ZnO	10 × 10-mm array	80 W 40 kHz, 300-W Xe lamp	95% 75 min	RhB	5	Xiang et al. (2020)
Ag_3_PO_4_/ZnO	30 mg	50 W 40 kHz, 300-W Xe lamp	98.16% 30 min	MB	-	Yu et al. (2020)
BiOI/ZnO	10 mg	90 W 40 kHz, 300-W Xe lamp	100% 30 min	BPA	10	Zhang et al. (2020a, b)
AgI/ZnO	20 mg	40 kHz, 250-W Xe lamp	91.9% 40 min	RhB	10	Liu et al. (2021a, b, c, d)
ZnO/MoS_2_	2 cm × 2 cm	Mechanical stress, 300-W Xe lamp	92.7% 50 min, 71.9% 50 min, 76.6% 80 min, 74.4% 25 min	MO, MB, CR, Cr(VI)	10, 5, 90, 10	Fu et al. (2021a, b)
ZnO/ZnS	–	360 W, UV lamp	60.7% 50 min	MB	5	Ren et al. (2021)
ZnO/ZnS/MoS_2_	10 mg	Mechanical agitator, 300-W Xe lamp	87.14% 50 min	MB	10	Fu et al. (2021a, c)
ZnO/CdS	25 mg	150 W, 300-W Xe lamp	100% 30 min	BPA	10	Zhang et al. (2021a, b)
BaTiO_3_/ZnO	60 mg	120 W, UV lamp	98.94% 90 min	RhB	5	Zheng et al. (2022a, b)
PVDF–ZnO/Cu	–	US, UV light	100% 90 min	RhB	-	Bootchanont et al. (2022)
Bi_2_WO_6_/g-C_3_N_4_/ZnO	30 mg	120 W 40 kHz, 100-W Xe lamp	98% 30 min	RhB	5	Kang et al. (2022)
PVDF/ZnO/Au	–	200 W, 300-W Xe lamp	100% 60 min	MO	20	Zhao et al. (2022)
ZnO/Fe_3_O_4_	40 mg	US, 175-W UV	99% 80 min	RhB	5	Zhang et al. (2022a, b)
ZnO hollow pitchfork	50 mg	120 W 40 kHz, 100-W Xe lamp	85% 80 min	RhB	10	Sharma et al. (2022a, b)
BaTiO_3_//ZnO/PVDF	50 mg	800-rpm stir, 300-W Xe lamp	91.05% 60 min, 90.12% 60 min, 96.33% 60 min, 93.65% 60 min	BPA, CR, MB, TCH	10	Lv et al. (2023)
ZnO/CuS	400 mg L^−1^	120 W, 300-W Xe lamp	85.28% 60 min	TCH	30	Gao et al. (2023a, b)
ZnO/g-C_3_N_4_-Ni foam	–	Mechanical agitator, 300-W Xe lamp	93.7% 120 min	MB	10	Ren et al. (2023)
ZnO	2.5 mg	140 W 35 kHz, LOT-Oriel Solar S class A solar simulator	50% 45 min	Testosterone	14.421	Bettini et al. (2023)
Al-ZnO	25 mg	100 W 40 kHz, 50-W lamp	76.6% 150 min	MB	25	Manoharan et al. (2023)

## The piezo-photocatalytic XTiO_3_-based materials

### Introduction of XTiO_3_

PZT (PbZr_1−*x*_Ti_*x*_O_3_) piezoelectric ceramics have been widely utilized in piezoelectric actuator technology for over 70 years, serving as the dominant choice in this field (Haertling 1999). Nevertheless, the Pb-based material family is currently encountering environmental compatibility issues due to the presence of the toxic heavy-metal element Pb. This poses risks during manufacturing, utilization, and disposal processes. Furthermore, stringent global regulations now demand the removal of Pb from all consumer products, creating an immediate necessity to develop a Pb-free alternative that can replicate the piezoelectric properties of the materials containing Pb (Wang et al. 2013; Wu et al. 2015; Zhang et al. 2015; Zhang et al. 2006; Zheng et al. 2017). While the majority of Pb-free materials currently available demonstrate piezoelectric properties that are inferior to those of Pb-based materials (Li et al. 2013; Rödel et al. 2009; Shrout and Zhang 2007; Takenaka and Nagata 2005). Solid-state physicists and material scientists have persisted in their pursuit of environmentally friendly piezoelectric materials, as evidenced by the increasing number of publications dedicated to Pb-free alternatives in recent decades.

### Introduction of BaTiO_3_

BaTiO_3_, belonging to the perovskite family, has garnered significant attention due to its remarkable properties, including a high dielectric constant and excellent ferroelectric properties. This intriguing material offers advantages such as biocompatibility, piezoelectric properties, and non-linear optical features, making it a promising candidate for various applications (Genchi et al. 2016). Additionally, comparative studies have reported the superior frequency response and power generation capabilities of BaTiO_3_ when compared to ZnO and barium sodium niobite (BNN) (Zaki et al. 2021).

### Degradation of MO dye

In a study conducted by Xu et al. (Xu et al. 2019a, b), Au nanoparticles were selectively deposited onto piezoelectric BaTiO_3_ nanocubes. The researchers conducted a thorough investigation of the degradation of MO using this system. Remarkably, the results demonstrated the complete degradation of MO within a mere 75 min when subjected to full-spectrum light irradiation, aided by auxiliary ultrasonic excitation. Furthermore, in a separate study conducted by Liu et al. (Liu et al. 2020), BaTiO_3_ nanowires were synthesized utilizing a two-step hydrothermal method. The researchers explored the potential of these nanowires for the degradation of MO at a concentration of 5 mg/L. Remarkably, it was observed that MO could be degraded by an impressive 98.17% within a span of 80 min by employing the synergistic effect of 180-W 40-kHz ultrasonicate vibrations and a UV lamp. In a separate noteworthy investigation, Fu et al. (2022) successfully employed the hydrothermal method to fabricate BaTiO_3_, which was further utilized to construct BaTiO_3_@TiO_2_ hybrid nanofibers through sol–gel assisted electrospinning. In their experimental setup, the researchers effectively achieved the complete degradation of MO dye within a remarkably short duration of 60 min. This outstanding result was accomplished by harnessing the combined effects of 180-W 40-kHz ultrasonic vibrations and a 160-W UV lamp, which synergistically enhanced the degradation process.

### Degradation of multiple pollutants

In a recent study, Xiong et al. (Xiong et al. 2023) investigated the degradation of nitenpyram, a pesticide, by a composite of AgI/Ag_3_PO_4_/BaTiO_3_. The study was conducted in aqueous solution, and the degradation efficiency of nitenpyram was found to be 100% after 10 min of reaction time. The authors attributed the high degradation efficiency to the synergistic effects of the three components of the composite, as well as the use of 200-W 40-kHz ultrasonic vibrations and a 300-W Xe lamp. Gao et al. (2023a, b) recently conducted experiments to investigate the efficiency of Ce-doped BaTiO_3_ in the degradation of multiple dyes. The study found that Ce-doped BaTiO_3_ was able to degrade Acid Fuchsin (AF), Crystal Violet (CV), and CR with high efficiency, with degradation rates of 99.1%, 99.4%, and 88.6%, respectively. These results were obtained under conditions of 10-min time, 50-W 80-kHz ultrasonic vibrations, and 300-W Xe lamp. In a separate investigation carried out by S. Wang and colleagues (Wang et al. 2023a, b), they successfully created a nanocomposite comprising BaTiO_3_/g-C_3_N_4_. Through a series of experiments focused on levofloxacin, the team assessed the nanocomposite’s efficacy in degrading the substance. Remarkably, within a mere 20-min span and under the influence of 100-W ultrasonic vibrations and a 300-W Xe lamp, the nanocomposite achieved an impressive degradation efficiency of 90.5% for levofloxacin. This breakthrough underscores the potent capabilities of this composite in rapidly breaking down pollutants. The team led by S. Gong. (Gong et al. 2023) achieved a significant advancement by producing the BaTiO_3_/g-C_3_N_4_ composite, a remarkable dual-functional photocatalyst with piezoelectric properties. In their innovative work, this composite showcased exceptional performance as a piezoelectric photocatalyst. For instance, when subjected to a 300-W Xe lamp and 200-W 40-kHz ultrasonic vibrations, it efficiently degraded tetracycline hydrochloride by an impressive 91.0% within a brief 60-min timeframe. This breakthrough underscores the composite’s exceptional potential in the realm of environmentally friendly pollutant degradation. In a groundbreaking endeavor, H. Lv and collaborators (Lv et al. 2023) synthesized Janus nanofibers composed of BaTiO_3_ and ZnO. This innovative composite exhibited remarkable capabilities in addressing diverse pollutants, including bisphenol A, Congo Red, methylene blue, and tetracycline hydrochloride. Through a combination of stirring and exposure to a potent 300-W Xe lamp, these nanofibers showcased their prowess in pollutant degradation. Notably, the removal rates were astonishingly high, with BPA being reduced by 94.75%, CR by 93.45%, MB by 99.06%, and TCH by 97.65%, all achieved within a mere 60-min period. This breakthrough study underscores the exceptional potential of the BaTiO_3_//ZnO Janus nanofibers in efficiently purifying diverse contaminants from various sources. Wan and the team. (Wan et al. 2023) achieved a remarkable feat by creating a core–shell structure of barium titanate within a COF, referred to as BTO@TD-COF. This ingenious composite capitalizes on its dual strengths of effective catalytic action and exceptional adsorption capabilities. Notably, these attributes enable the composite to achieve transformative results in pollutant treatment. For instance, the pernicious BPA is eradicated entirely, while 2,4-dichlorophenol (2,4-DCP) experiences an 86.8% reduction, and phenol is notably diminished by 59% within a relatively brief 75-min duration. These impressive outcomes are attained through the strategic implementation of 180-W 40-kHz ultrasonic vibrations and a 200-W Xe lamp. This study underscores the immense potential of the BTO@TD-COF core–shell composite in efficiently neutralizing a spectrum of pollutants, underscoring its significance in advancing sustainable environmental solutions. Masekela and colleagues (Masekela et al. 2023a, b) achieved a significant breakthrough by producing a thin film composed of fluorine-doped tin oxide/barium titanate (FTO/BTO) loaded with SnO_2_. In addition to its innovative fabrication, this film was subjected to rigorous testing in the degradation of various organic pollutants, namely, MO, MB, and CIP. The outcomes were notably impressive, with degradation rates reaching 94%, 92%, and 64%, respectively, for these pollutants. These experiments were meticulously conducted under the influence of 40-kHz ultrasonic vibrations paired with the power of a 100-W Xe lamp. This study not only highlights the advanced composite material’s potential, but also underscores its capability to efficiently combat a range of pollutants through its adept combination of ultrasonic and photocatalytic effects (Table 2).

**Table 2:** Performance of XTiO_3_-based composites with different effluents

Catalyst	Catalyst dosage	Condition	Performance	Effluent	Effluent concentration (mg L^−1^)	Reference
Ba_1−*x*_Ca_*x*_TiO_3_	100 mg	120 W 40 kHz, UV lamp	100% 40 min	MO	5	Lin et al. (2019)
AuNPs/BaTiO_3_	50 mg	Ultrasonic + 300-W Xe lamp	100% 75 min	MO	10	Xu et al. (2019a, b)
Ag/BaTiO_3_	50 mg	Ultrasonic + full light spectrum	91% 75 min	RhB	10	Xu et al. (2019a, b)
Ag-Ag_2_S/BaTiO_3_	50 mg	Ultrasonic + full light spectrum	90% 30 min	MO	3.273	Lei et al. (2020)
Na_0.5_Bi_0.5_TiO_3_	200 mg	120 W 40 kHz, 500-W Xe lamp	98% 180 min	RhB	10	Zhang et al. (2020a, b)
Bi_0.5_Na_0.5_TiO_3_@TiO_2_	500 mg L^−1^	100 W 40 kHz, 500-W Xe lamp	97% 90 min	RhB	10	Xu et al. (2020)
Rh-doped SrTiO_3_	6 mg	150 W 40 kHz, 300-W Xe lamp	98.3% 15 min	BPA	10	Zhou et al. (2020)
BaTiO_3_ nanowires	–	180 W 40 kHz, UV-LED	98.17% 80 min	MO	5	Liu et al. (2020)
BaZr_0.02_Ti_0.98_O_3_	100 mg	70 W 40 kHz, 24-W UV lamp	89% 240 min	RhB	6	Sharma et al. (2020)
Ba_0.85_Ca_0.15_Ti_0.9_Zr_0.1_O_3_	–	70 W 40 kHz, 8-W UV lamp	89% 180 min	RhB	-	Sharma et al. (2020)
_0.65_Pb(Mg_1/3_Nb_2/3_)O_3_-_0.35_PbTiO_3_/SnO_2_	50 mg	200 W 45 kHz, 250-W metal halide lamp	100% 70 min	MB	20	Dursun et al. (2021)
BaTiO_3_/La_2_Ti_2_O_7_	–	210 W 40 kHz, 300-W Xe lamp	50.2% 90 min	CIP	10	Li et al. (2021)
Ag/BaTiO_3_	–	300 W US, 500-W Xe lamp	48.9% 100 min	MB	10	Fu et al. (2021a, b)
Ag-BaTiO_3_	100 mg	70 W 40 kHz, 15-W LED	96% 180 min	RhB	5	He et al. (2021)
Bi_0.5_Na_0.5_TiO_3_@BiVO_4_	50 cm^2^	US, 300-W Xe lamp	80% 100 min	RhB	10	Liu et al. (2021a, b, c, d)
BaTiO_3_/KNbO_3_	10 mg	45 kHz, 300-W Xe lamp	93.3% 180 min	Direct Lake Blue 5B	–	Zhang et al. (2021a, b)
_0.3_Ba_0.7_Ca_0.3_TiO_3_-_0.7_BaSn_0.12_Ti_0.88_O_3_	50 mg	210 W 40 kHz, 300-W Xe lamp	90% 60 min, 95% 60 min	MB, RhB	5, 6	Raj et al. (2021)
(Na_0.5_Bi_0.5_)TiO_3_-Ba(Ti_0.5_Ni_0.5_)O_3_	1 g L^−1^	200 W 40 kHz, 300-W Xe lamp	100% 20 min	RhB	10	Xiao et al. (2021)
Li/La-doped BaTiO_3_	100 mg	Ultrasonic, 300-W Xe lamp	95% in 12 min	RhB	5	Yu et al. (2022a)
Ag/_0.5_(Ba_0.7_Ca_0.3_)TiO_3_-_0.5_Ba (Zr_0.1_Ti_0.9_)O_3_	100 mg	70 W 40 kHz, 30-W Havells lamp	96% 90 min	MB	5	Sharma (2022)
BaTiO_3_/ZnO	60 mg	120-W US, UV lamp	98.94% 90 min	RhB	5	Zheng et al. (2022a, b)
Bi_0.5_Na_0.5_TiO_3_ @Ag	10 mg	120 W 40 kHz, 300-W Xe lamp	83.5% 90 min, 95.3% 40 min, 96.8% 80 min	CIP, MO, mitoxantrone hydrochloride	10	Wang et al. (2022c)
Ag@5Na_0.5_Bi_0.5_TiO_3_	20 mg	300 W 40 kHz, 300-W Xe lamp	98.8% 30 min	RhB	5	Shi et al. (2022)
Ag-BaTiO_3_	10 mg	120 W 24 kHz, 300-W Xe lamp	82.7% 120 min	MO	10	Chen et al. (2022a, b)
BaTiO_3_/CuO	100 mg	US, 200-W Xe lamp	90% 45 min	MO	10	Yu et al. (2022a)
NiO@PbTiO_3_	25 mg	300 W 40 kHz, 6-W UV LED	95% 6 min	RhB	5	Xie et al. (2022)
Al-doped SrTiO_3_/TiO_2_	20 × 30-mm nanorod array	100 W 40 kHz, 100-mW/cm^2^ visible	100% 120 min	RhB	5	Chu et al. (2022)
Na_0.5_Bi_0.5_TiO_3_	50 mg	40 kHz, 300-W Xe lamp	100% 80 min	RhB	10	Ji et al. (2022)
BaTiO_3_/Ti_3_C_2_T_*x*_	100 mg	100 W 40 kHz, 300-W Xe lamp	94.3% 60 min	Phenol	20	Zheng et al. (2022a, b)
BaTiO_3_@TiO_2_	50 mg	180 W 40 kHz, 160-W UV lamp	100% 60 min	MO	5	Fu et al. (2022)
BaTiO_3_/AgAlO_2_	50 mg	40 kHz, 300-W Xe lamp	90% 90 min	MB	~ 80	Hou et al. (2022)
BaTiO_3_/WS_2_	–	300 W 40 kHz, yellow LED lamp	90% 75 min	MO	-	Fazli (2022)
PVDF@BT/MoS_2_/Au	–	100 W 40 kHz, 400-W Xe lamp	99.7% 10 min, 97.6% 60 min	MB, OTC	10	Shan et al. (2022)
BaTiO_3_@TiO_2_	50 mg	45 kHz, 300-W Xe lamp	99.7% 20 min	RhB	10	Liu et al. (2022a, b)
FTO/BaTiO_3_/AgNPs	2 cm × 2 cm	30 W 24 kHz, 70-W LD3001	72% 180 min, 98% 180 min	CIP, MB	5	Masekela et al. (2022)
Bi_0.5_Na_0.5_TiO_3_/PVDF	100 mg	200 W 45 kHz, 300-W Xe lamp	90.8% 180 min	RhB	5	Zhou et al. (2022a, b)
FTO/BaTiO_3_/SnO_2_	–	40 kHz, 100-W Xe lamp	94% 180 min, 92% 180 min, 64% 180 min	MO, MB, CIP	5	Masekela et al. (2023a, b)
Ba_0.85_Ca_0.15_Zr_0.1_(Ti_1−*x*_Co_*x*_)0.9O_3_	100 mg	200 W 100 kHz, 300-W Xe lamp	99.1% 60 min	RhB	10	Zhao et al. (2023)
AgI/Ag_3_PO_4_/BaTiO_3_	10 mg	200 W 40 kHz, 300-W Xe lamp	100% 10 min	Nitenpyram	5	Xiong et al. (2023)
_0.5_Ba(Zr_0.2_Ti_0.8_)O_3_-_0.5_(Ba_0.7_Sr_0.3_) TiO_3_	100 mg	150 W 40 kHz, 30-W LED lamp	92% 240 min	MB	5	Dubey et al. (2023)
Ce-doped BaTiO_3_	100 mg	50 W 80 kHz, 300-W Xe lamp	99.1% 10 min, 99.4% 10 min, 88.6% 10 min	AF, CV, CR	10	Gao et al. (2023a, b)
Ag-Na_0.5_Bi_0.5_TiO_3_	100 mg	50 W 40 kHz, 300-W Xe lamp	98.6% 120 min	RhB	5	Shi et al. (2023)
Pt/BaTiO_3_	20 mg	100 W 53 kHz, 300-W Xe lamp	92.5% 50 min	MO	10	Meng et al. (2023)
BaTiO_3_/g-C_3_N_4_	10 mg	100-W US, 300-W Xe lamp	90.5% 20 min	Levofloxacin	20	Wang et al. (2023a, b)
BaTiO_3_/g-C_3_N_4_	20 mg	200 W 40 kHz, 300-W Xe lamp	91% 60 min	TCH	10	Gong et al. (2023)
Ag NWs@BaTiO_3_	50 mg	80-W US, 10-W lamp	92.5% 60 min, 100% 30 min	*S. aureus*, *E. coli*	10	Shu et al. (2023)
BaTiO_3_//ZnO/PVDF	50 mg	800-rpm stir, 300-W Xe lamp	91.05% 60 min, 90.12% 60 min, 96.33% 60 min, 93.65% 60 min	BPA, CR, MB, TCH	10	Lv et al. (2023)
BaTiO_3_ @TD-COF	5	180 W 40 kHz, 200-W Xe lamp	100% 75 min, 86.8% 75 min, 59% 75 min	BPA, 2,4-DCP, phenol	20	Wan et al. (2023)

## The piezo-photocatalytic bismuth-based materials

### Introduction on BiVO_4_

Bismuth-based compounds such as BiVO_4_ show potential as a photocatalyst due to its narrow band gap (2.4–2.5 eV), reduced environmental risks, and cost-effectiveness (Cooper et al. 2014). The monoclinic form of bismuth vanadate, referred to as m-BiVO_4_, exhibits distinct attributes such as solar energy absorption, ferroelastic behavior, ionic conduction, and the ability to produce hydrogen (Tahir et al. 2019; Wang et al. 2014). Researchers have discovered that BiVO_4_ demonstrates localized surface piezoelectric properties, rendering it appropriate for employment in piezocatalysis (Munprom et al. 2014).

### The work with bismuth-based material

Liu and colleagues (Liu et al. 2021a, b, c, d) achieved a significant feat by creating environmentally friendly piezoelectric materials, namely, Bi_0.5_Na_0.5_TiO_3_@BiVO_4_, through a hydrothermal synthesis method. They systematically evaluated the degradation performance of these materials under the influence of ultrasonic waves and a potent 300-W Xe lamp. Impressively, their innovative approach led to an 80% degradation of RhB within a 100-min span. This groundbreaking research not only highlights the potential of their Pb-free piezoelectric materials but also underscores their capability to efficiently combat pollutants through the strategic application of ultrasonic vibrations and advanced light sources. M. Kumar and collaborators (Kumar et al. 2022a) achieved a notable advancement through the creation of BiVO_4_ using the mechanochemical high-energy ball milling technique. Their study encompassed a comprehensive exploration of the material’s capabilities in both photocatalysis and piezocatalysis. Particularly intriguing was their investigation into the combined piezoelectric and photocatalytic effects. One remarkable result was the application of this synergy to degrade MB dye, showcasing a significant 81% reduction within a span of 240 min. This innovative research highlights the potential of their fabricated BiVO_4_, not only as a standalone photocatalyst but also in harnessing the combined power of piezoelectric and photocatalytic phenomena for effective pollutant degradation. The team led by Wang (Wang et al. 2022a, b, c, d, e) achieved a significant breakthrough by developing a layered structure composed of 2D Bi_4_Ti_3_O_12_–BiVO_4_–Bi_4_V_2_O_10_, utilizing a hydrothermal synthesis approach. Their exploration extended to two crucial aspects: the effective removal of Cr(VI) and the facilitation of oxygen evolution, both under the influence of both light and ultrasound treatments. In a remarkable demonstration of their innovative material, they succeeded in entirely eliminating Cr(VI) within a mere 50 min. This impressive feat was achieved through the strategic combination of 200-W 40-kHz ultrasonic vibrations and the powerful output of a 300-W Xe lamp. This study not only highlights the potential of their engineered structure but also showcases its dual prowess in addressing environmental contaminants and driving oxygen generation, thereby contributing significantly to sustainable technological advancements.

In a groundbreaking effort, Deka and the team (Deka et al. 2022) successfully synthesized BiVO_4_ nanorods using a hydrothermal technique. Their research extended to the practical application of these nanorods, particularly in the context of pollutant degradation. The team systematically examined the material’s capabilities by subjecting it to the dual influence of light and ultrasound vibrations. Impressively, their findings revealed a rapid degradation rate of 97.13% for methylene blue in a remarkably short 40-min timeframe. This exceptional result was achieved by synergistically employing 35-kHz 20-W ultrasonic vibrations and the illuminating power of an LED lamp. This study not only underscores the potential of their fabricated nanorods but also signifies their capacity to efficiently address pollution concerns through the innovative fusion of ultrasonic and photocatalytic effects. In an innovative endeavor, T. Wu and co-researchers skillfully engineered tubular carbon nitride derived from fish scales, labeled as FTCN. Moreover, they ventured into the creation of a composite, blending V-BiOIO_3_ with FTCN, aiming to explore its potential in the realm of piezo-photocatalysis. Their focus was on TCH, a significant pollutant. Impressively, their efforts yielded remarkable results, showcasing an impressive degradation rate of 89% within a mere 60-min span. This achievement was realized through a strategic pairing of 200-W ultrasonic vibrations and the intense illumination provided by a 300-W Xe lamp. This study not only underscores the ingenuity behind FTCN and V-BiOIO_3_/FTCN composites but also highlights their exceptional ability to synergistically harness ultrasonic and photocatalytic effects, paving the way for efficient pollutant mitigation techniques. In a notable achievement, Lu and colleagues (Lu et al. 2023) successfully synthesized Na_0.5_Bi_4.5_Ti_4_O_15_, a novel material harnessed for the degradation of RhB. Their research embarked on the investigation of this material’s potential in environmental purification. Strikingly, their diligent efforts, combined with the orchestrated effects of 180-W 40-kHz ultrasonic vibrations and the illuminating strength of a 300-W Xe lamp, yielded a remarkable outcome—a full 100% efficiency in dismantling the targeted organic dye. This pivotal accomplishment not only underscores the proficiency of Na_0.5_Bi_4.5_Ti_4_O_15_ but also illuminates its capability as a robust tool in the realm of pollutant eradication. This achievement, achieved within a concise 90-min timeframe, accentuates the material’s rapid and effective pollutant-degrading potential through the synergistic interplay of ultrasonic and photocatalytic processes. In a significant accomplishment, Ma and the team (Ma et al. 2023) achieved the degradation of sulfamethoxazole by an impressive 86% within a mere 40-min span. This remarkable feat was realized through the synergistic action of 53-kHz ultrasonic vibrations and the intense irradiation of a 300-W Xe lamp. Central to this achievement was the production of Bi_2_WO_6_ nanosheets, synthesized using the hydrothermal method. This innovative approach highlights the potential of Bi_2_WO_6_ nanosheets as a potent tool in environmental remediation, showcasing their remarkable ability to rapidly degrade pollutants such as sulfamethoxazole. The combination of ultrasonic and photocatalytic effects underscores the effectiveness of this novel approach in addressing waterborne contaminants efficiently and swiftly.

Li and colleagues (Li et al. 2023a, b, c) accomplished a significant achievement through the hydrothermal synthesis of BiOCl. Their innovative endeavor extended to the removal of a diverse array of pollutants, including RhB, MO, CR, and MB. Remarkably, their approach led to highly effective degradation, yielding efficiency rates of 96.91%, 79.8%, 72.2%, and 38.3%, respectively. All this was accomplished astonishingly within a mere 3-min timeframe, under the simultaneous influence of 100-W 40-kHz ultrasonic vibrations and the potent illumination of a 300-W Xe lamp. This groundbreaking study not only underscores the prowess of BiOCl but also showcases its exceptional ability to swiftly and effectively address a spectrum of pollutants. The synergistic utilization of ultrasonic and photocatalytic mechanisms underscores the rapid and efficient nature of this method, enhancing its significance in environmental purification endeavors (Table 3).

**Table 3:** Performance of bismuth-based composites with different effluents

Catalyst	Catalyst dosage	Conditions	Performance	Effluent	Effluent concentration (mg L^−1^)	References
Bi_0.5_Na_0.5_TiO_3_ @BiVO_4_	50 cm^2^	US, 300-W Xe lamp	80% 100 min	RhB	10	Liu et al. (2021a, b, c, d)
Bi_2_MoO_6_/BiOBr	50 mg	US, 400-W metal halide lamp	99.6% 60 min	Methyl violet	20	Yao et al. (2021)
Bi_4_Ti_3_O_12_	–	100 W 40 kHz, 175-W Xe lamp	100% 56 min	RhB	5	Liu et al. (2021a, b, c, d)
CNT/Bi_4_O_5_I_2_	50 mg	80 W 40 kHz, 300-W Xe lamp	91% 80 min	RhB	5	Wang et al. (2021)
NaNbO_3_/CuBi_2_O_4_	50 mg	30-W US, 50-W LED lamp	75% 90 min	RhB	10	Dilly Rajan et al. (2021)
Bi_2_WO_6_/g-C_3_N_4_/ZnO	30 mg	120 W 40 kHz, 100-W Xe lamp	98% 30 min	RhB	5	Kang et al. (2022)
BiVO_4_	200 mg	US, 30-W Havells lamp	81% 240 min	MB	5	Kumar et al. (2022a)
Pt/Bi_3.4_Gd_0.6_Ti_3_O_12_	20 mg	100 W 53 kHz, 300-W Xe lamp	92% 70 min	MO	10	Liang et al. (2022)
Bi_4_Ti_3_O_12_–BiVO_4_–Bi_4_V_2_O_10_	50 mg	200 W 40 kHz, 300-W Xe lamp	100% 50 min	Cr(VI)	–	Wang et al. (2022a, b, c, d, e)
CuBi_2_O_4_	50 mg	300 W 40 kHz, 300-W Xe lamp	98.1% 80 min	RhB	5	Cao et al. (2022)
SrBi_4_Ti_4_O_15_/BiOCl	50 mg	180 W 40 kHz, 300-W Xe lamp	100% 7 min	RhB	5	Jia et al. (2022)
BiOIO_3_/basic bismuth (III) nitrate (BBN)	50 mg	180 W 40 kHz, 300-W Xe lamp	100% 3 min, 90.86% 3 min, 88.72% 3 min, 89.64% 3 min	RhB, CR, MO, MB	10	Li et al. (2022a, b)
Bi_0.5_Na_0.5_TiO_3_/MWCNTs	50 mg	120 W 40 kHz, 300-W Xe lamp	90% 30 min	RhB	5	Wang et al. (2022d)
SrBi_4_Ti_4_O_15_	500 mg L^−1^	100-W US, visible light	98% 70 min	TC	44.4	Zhu et al. (2022)
Au-BiOBr	1000 mg L^−1^	100 W 40 kHz, 300-W Xe lamp	95.8% 30 min	CBZ	5	Hu et al. (2022a, b)
Bi_2_WO_6_	500 mg L^−1^	120 W 40 kHz, LED lamp	100% 40 min	RhB	60	Jiang et al. (2022a, b)
Bi_3.25_La_0.75_Ti_3_O_12_	70 mg	US, 300-W Xe lamp	93.04% 60 min, 96.3% 12 min	2,4-DCP, TC	15	Zhong et al. (2022)
Bi_2_VO_5.5_	250 mg	150 W 40 kHz, 15-W Havells lamp	82% 240 min	MB	5	Kumar et al. (2022)
Bi_2_WO_6_/black–TiO_2_	20 mg	240-W US, 220-W Xe lamp	98.43% 60 min	RhB	10	Shen et al. (2022)
Bi_2_WO_6_	1000 mg L^−1^	180 W 40 kHz, 400-W metal halide lamp	98.39% 70 min	RhB	10	Hu et al. (2022a, b)
BiOCl/NaNbO_3_	100 mg	50 W 40 kHz, 300-W Xe lamp	87.4% 100 min	RhB	5	Li et al. (2022a, b)
Ti-BiOCl	70 mg	US, 300-W Xe lamp	93.97% 10 min	TC	15	Liu et al. (2022a, b)
Ti_3_C_2_-BiOBr	60 mg	100 W 35 kHz, 400-W metal halide lamp	99.8% 48 min	Methyl violet	10	Yao et al. (2022)
BiVO_4_	30 mg	100 W 40 kHz, 300-W Xe lamp	97% 40 min	MB	3	Wang et al. (2022a, b, c, d, e)
BiVO_4_	50 mg	35 kHz, 20-W LED lamp	97.13% 40 min	MB	2	Deka et al. (2022)
CaBiO_3_	90 mg	100 W 40 kHz, 150-W lamp	94% 120 min	Cr(VI)	–	Khosya et al. (2022)
BaBi_4_Ti_4_O_15_	100 mg	150-W US, 24-W UV lamp	62% 120 min	MB	5	Kumar et al. (2023)
Cu/Na_0.5_Bi_4.5_Ti_4_O_15_	100 mg	Magnetic stirrer, 300-W Xe lamp	96% 120 min	MO	10	Lan et al. (2023)
BiOCl	50 mg	100 W 40 kHz, 300-W Xe lamp	96.91% 3 min, 79.8% 3 min, 72.2% 3 min, 38.3% 3 min	RhB, MO, CR, MB	10	Li et al. (2023a, b, c)
Bi_2_NdO_4_Cl	20 mg	Mechanical stress, 500-W Xe lamp	39.2% 120 min	TCH	10	Lin et al. (2023)
SrBi_4_Ti_4_O_15_/Ag_2_O	50 mg	180 W 40 kHz, 300-W Xe lamp	100% 6 min	RhB	5	Jia et al. (2023)
Bi_2_WO_6_	20 mg	53 kHz, 300-W Xe lamp	86% 40 min	Sulfamethoxazole	25	Ma et al. (2023)
Bi_4_Ti_3_O_12_	80 mg	120 W 40 kHz, 300-W Xe lamp	100% 15 min	RhB	5	Tang et al. (2023)
V-BiOIO_3_/FTCN	10 mg	200-W US, 300-W Xe lamp	89% 60 min	TCH	10	Wu et al. (2023)
Bi_4_Ti_3 − 2*n*_Cr_*n*_Nb_*n*_O_12_/g-C_3_N_4_	30 mg	45 kHz, 300-W Xe lamp	98.7% 45 min	RhB	10	Bai et al. (2023)
Bi_4_Ti_3_O_12_	50 mg	70 W 40 kHz, mercury lamp	86% 120 min	RhB	10	Liu et al. (2023)
Na_0.5_Bi_4.5_Ti_4_O_15_	–	180 W 40 kHz, 300-W Xe lamp	100% 90 min	RhB	–	Lu et al. (2023)

## Water splitting and hydrogen evolution

### Energy demand and hydrogen

**Fig. 5:**
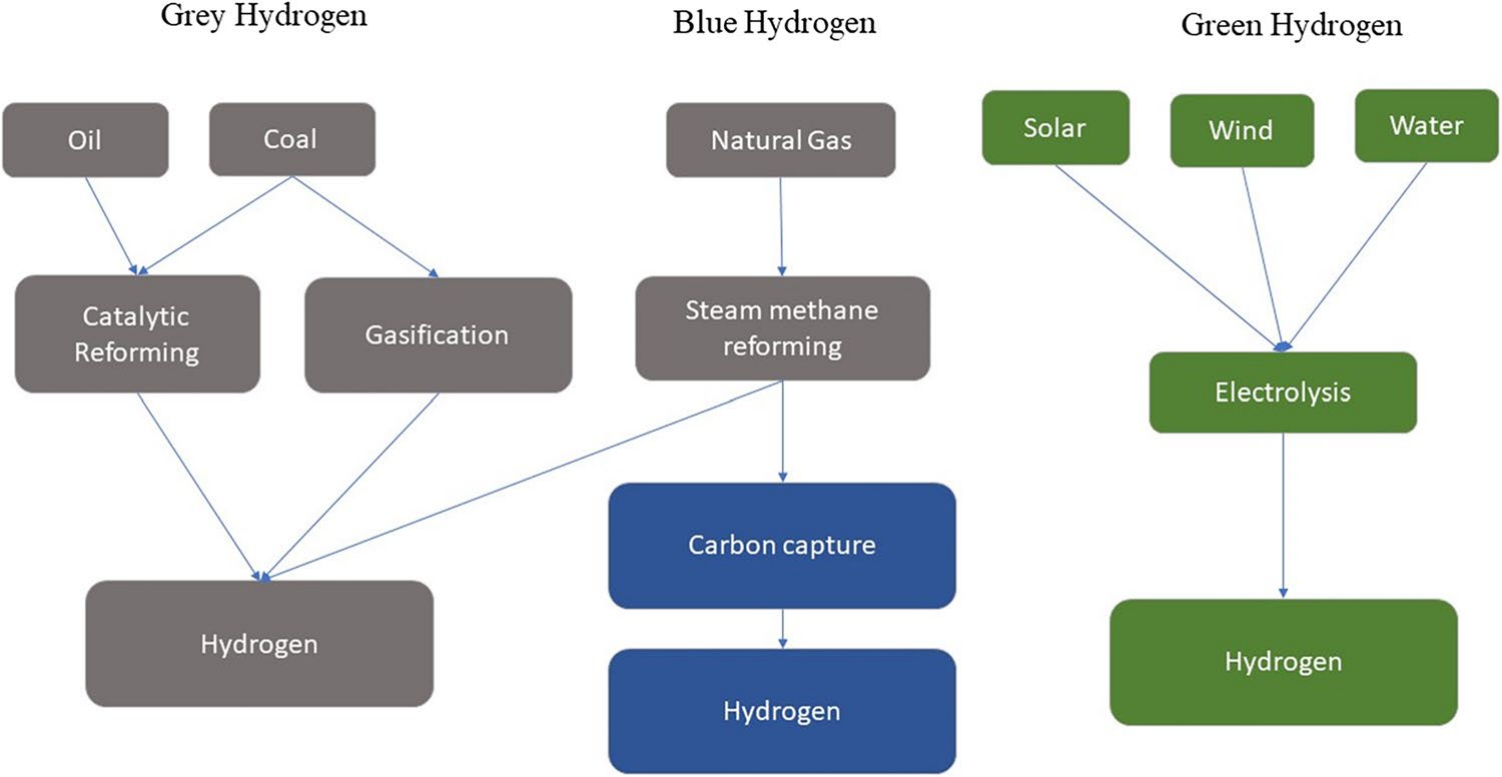
The hydrogen production processes

In the face of continually rising energy demands and pressing environmental concerns, it becomes imperative to formulate methodologies that efficiently harness the potential of renewable natural resources (Garcia-Sanchez et al. 2019). The recent culmination of the United Nations conference on climate change, known as COP26, held in Glasgow in November 2021, underscored the imperative of transitioning towards clean energy sources. The focus on transitioning away from coal and other fossil fuels particularly underscores the urgency of creating sustainable, low-carbon energy pathways for the times ahead. This marks a significant step towards addressing the global need for environmental responsibility and sustainable energy solutions (“COP26: green technologies could turn the tide,” 2021). In this context, green hydrogen emerges as a favorable clean energy carrier, attributed to its emission-free properties, and holds the capacity to supplant traditional fossil fuel reservoirs (Hanley et al. 2018; Staffell et al. 2019). Currently, hydrogen predominantly originates from the steam methane reforming procedure, widely recognized as “gray hydrogen” (Yao et al. 2019). Nonetheless, this method exerts a substantial ecological toll by releasing a significant amount of CO_2_ (ranging from 9 to 12 kg CO_2_ per kg of H_2_) and is also dependent on a consistent methane source (typically derived from fossil fuel origins). This not only accentuates environmental concerns but also underscores the reliance on non-renewable resources (Sun et al. 2019). In efforts to address the carbon emissions associated with gray hydrogen manufacturing, avenues involving carbon capture and storage have been investigated, thereby laying the foundation for the emergence of “Blue Hydrogen” (Fig. 5).

### Green hydrogen

H_2_ derived through water splitting, often referred to as green hydrogen, holds a crucial status as a sustainable energy source. This particular resource has captured the enduring attention of the scientific realm owing to its notable energy density and eco-friendly characteristics (Gautam et al. 2023; Sk et al. 2022; Vennapoosa et al. 2023; Xu et al. 2021a, b). This necessity has spurred the advancement of effective methods for converting H_2_ in an environmentally friendly manner. Presently, water splitting primarily relies on electrocatalysis; however, there is growing enthusiasm for exploring alternative routes like photocatalysis and piezocatalysis. These approaches hold considerable promise in achieving H_2_ production through the utilization of sustainable energy sources, namely, solar energy and natural environmental vibrations. The quest for efficient and eco-conscious hydrogen conversion processes is driven by the urgency of a cleaner energy landscape (Liu et al. 2016; Pan et al. 2020; Wang et al. 2019; Wang and Domen 2020). However, when it comes to essential practical applications in industries, these individual catalytic methods often encounter restrictions concerning their economic viability, overall efficiency, and long-term stability. This highlights the significant challenges that need to be addressed for these methods to be effectively integrated into industrial contexts (Ran et al. 2020; Tu et al. 2020; Wei et al. 2019). Therefore, novel approaches to address the previous challenges are desirable.

### Work of others

In a pioneering effort, Yang and colleagues (Yang et al. 2022) introduced an innovative piezo-photoelectric catalyst termed as coaxial TiO_2_-BaTiO_3_-CuInS_2_ heterostructures. The focal point of their research encompassed assessing the catalyst’s prowess in H_2_ production. This was accomplished under the concerted influence of a 300-W Xe lamp and 40-kHz ultrasonic vibrations at 100 W, over a span of 4 h. The remarkable findings unveiled a peak hydrogen production rate of 117 µmol h^−1^ cm^−1^, specifically achieved when the ultrasonic power was set at 100 W. Intriguingly, it became evident that any subsequent escalation in ultrasonic power correspondingly led to a reduction in hydrogen generation. This intricate interplay of variables highlights the delicate balance required in optimizing piezo-photoelectric catalysis for efficient hydrogen production, setting the stage for further exploration and refinement in this exciting field. Xu and their collaborators (Xu et al. 2022) orchestrated a groundbreaking endeavor by ingeniously merging COFs and piezoelectric substances through robust covalent bonds, thus yielding a novel Z-scheme core@shell heterostructure piezo-photocatalyst, known as BiFeO_3_@TpPa-1-COF. The crux of their study revolved around the remarkable achievements enabled by this material. Astonishingly, the researchers achieved compelling rates of H_2_ and O_2_ production, specifically measuring 1416.4 and 708.2 μmol h^−1^ g^−1^, respectively. These impressive results were realized through the innovative coupling of ultrasonication with visible light irradiation, unveiling the potential of this advanced hybrid catalyst for efficient and sustainable hydrogen and oxygen generation. This research not only introduces a groundbreaking catalytic concept but also offers a glimpse into the exciting possibilities within the realm of piezo-photocatalysis. Jiang and their research team (Jiang et al. 2023) orchestrated an innovative initiative by crafting BaTiO_3 − *x*_ nanoparticles, specifically geared towards hydrogen production. The fruit of their labor materialized in a promising achievement: an encouraging production rate of 132.4 μmol h^−1^ g^−1^ was attained utilizing deionized (DI) water, all through the ingenious process of piezo-photocatalysis, and intriguingly, without the incorporation of a co-catalyst. In a stark comparison, a substantial rate of 48.7 μmol h^−1^ g^−1^ was accomplished when dealing with the complexities of natural seawater, despite the challenging influence of dissolved ions. This investigation serves as a catalyst in itself, introducing fresh perspectives for the large-scale, environmentally friendly generation of hydrogen, leveraging the abundance of natural resources. What sets this study apart is the utilization of a conventional piezoelectric material—readily available and often utilized—yet its effectiveness is not deterred by the presence of ions dissolved within seawater. This paves the way for a more sustainable approach to hydrogen production, capitalizing on conventional resources and opening doors to scalable and ecologically sound methods. In a trailblazing endeavor, Jiang and collaborators (Jiang et al. 2022a, b) engineered OH-modified SrTiO_3_, instigating a meticulous exploration into hydrogen production rates through the prism of piezo-photocatalysis. Their efforts bore fruits of astonishing magnitude: an exceptional hydrogen production rate of 701.2 μmol h^−1^ g^−1^ was realized. This accomplishment reverberates with significance, as it eclipses the hydrogen evolution process of STO under conventional photocatalysis by an astounding 5.3-fold. This study unfolds as more than a scientific pursuit; it unveils a pathway towards the future of nanomaterial engineering, one that capitalizes on functional group modifications for enhanced piezo-photocatalytic efficacy. This study serves as an illuminating beacon, showcasing the potential of harnessing functional group engineering to usher in a new era of energy-efficient and high-performance hydrogen generation. Yu and a team of researchers (Yu et al. 2022a) embarked on an innovative endeavor, crafting Li/La-doped BaTiO_3_ with the aim of unraveling its hydrogen production potential within the realm of piezo-photocatalysis. Impressively, their endeavors bore fruit in the form of a noteworthy production rate of (3700 μmol h^−1^ g^−1^), a feat attributed to the heightened light absorption characteristics of the material. This study extends beyond the confines of a mere investigation; it pioneers a strategic pathway based on the infusion of heterovalent ions, sparking a harmonious piezo-phototronic interplay that catalytically amplifies the material’s prowess. In essence, their study unravels the fusion of mechanics and light as a catalyst for transformative advancements, offering a glimpse into a future where materials are engineered to harness energy and facilitate processes with unprecedented efficiency (Table 4).

**Table 4:** Performance of different composites in the water splitting

Catalyst	Conditions	Performance	Reference
Bi_0.5_Na_0.5_TiO_3_	110-W 40-kHz ultrasonic + 300-W Xe lamp	154.6 μmol g^−1^ h^−1^	Zhao et al. (2020)
PbTiO_3_/CdS	100-W 40-kHz ultrasonic + 300-W Xe lamp	849.0 μmol g^−1^ h^−1^	Huang et al. (2021)
CuS/ZnO	50-W ultrasonic + 50-W Xe lamp	140 μmol h^−1^ cm^−2^	Zhao, Wang, and Du (2021a, b)
BaTiO_3_@MoSe_2_	300-W 40-kHz ultrasonic + 150-W Xe lamp	(4533 μmol g^−1^ h^−1^)	Guo et al. (2021)
(Na_0.5_Bi_0.5_)TiO_3_-Ba(Ti_0.5_Ni_0.5_)O_3_	200-W 40-kHz ultrasonic + 300-W Xe lamp	450 μmol g^−1^ h^−1^	Xiao et al. (2021)
TiO_2_-BaTiO_3_-CuInS_2_	100-W ultrasonic + 300-W Xe lamp	117 μmol g^−1^ cm^−1^	Yang et al. (2022)
Li/La-doped BaTiO_3_	Ultrasonic + 300-W Xe lamp	3700 μmol g^−1^ h^−1^	Yu et al. (2022b)
OH-SrTiO_3_	Ultrasonic + 300-W Xe lamp	701.2 μmol g^−1^ h^−1^	Jiang et al. (2022a, b)
BaTiO_3 − *x*_	100-W 40-kHz ultrasonic + 8-W UV lamp	132.4 μmol g^−1^ h^−1^	Jiang et al. (2023)

## Conclusion and outlooks

In conclusion, this work has journeyed through the intriguing realm of piezo-photocatalysis, illuminating its multifaceted applications in addressing the pressing concerns of effluent treatment and sustainable hydrogen production. We began by delving into the fundamental principles of piezoelectricity and photocatalysis, recognizing their pivotal roles in advancing environmental science and renewable energy solutions.

The exploration then extended to the diverse array of effluents, from dyes to antibiotics, highlighting the multifarious challenges posed by these pollutants to both environmental ecosystems and human health. The in-depth analysis shed light on the urgency of effective treatment methods and underscored the promise of piezo-photocatalysis as a versatile and efficient solution.

Our investigation further scrutinized various composite materials, including ZnO-based composites, XTiO_3_-based composites, and bismuth-based composites. These materials exhibited remarkable catalytic properties and tunability, offering a rich palette of options for tailoring treatment processes to specific effluent types.

Finally, we ventured into the realm of hydrogen production, recognizing piezo-photocatalysis as a key player in the quest for clean and sustainable energy sources. The discussion encompassed various types of hydrogen and the potential of this technology in unlocking the hydrogen economy.

As we conclude this journey, it is evident that piezo-photocatalysis stands at the intersection of innovation and sustainability, offering promising avenues for addressing the world’s most pressing environmental and energy challenges. The synergy between materials science, environmental engineering, and renewable energy research exemplifies the power of interdisciplinary collaboration in shaping a brighter, more sustainable future. While challenges remain, the prospects are undeniably exciting, and further research in this field holds the key to transformative solutions that can benefit both our planet and future generations.

The future of piezo-photocatalysis research holds the promise of cleaner water, sustainable energy, and a healthier planet. As we delve deeper into the synergy between piezoelectricity and photocatalysis, and as we continue to innovate in materials science and catalytic engineering, we are poised to unlock transformative solutions that address some of the most pressing challenges of our time. The journey ahead will be marked by collaboration, discovery, and the pursuit of a more sustainable and environmentally conscious world.
